# Novel human genetic variants associated with extrapulmonary tuberculosis: a pilot genome wide association study

**DOI:** 10.1186/1756-0500-4-28

**Published:** 2011-01-31

**Authors:** Noffisat O Oki, Alison A Motsinger-Reif, Paulo RZ Antas, Shawn Levy, Steven M Holland, Timothy R Sterling

**Affiliations:** 1 Bioinformatics Research Center, Department of Statistics, North Carolina State University, Raleigh, NC, USA; 2 Division of Infectious Diseases, Department of Medicine, Vanderbilt University School of Medicine, Nashville, TN, USA; 3 Department of Biomedical Informatics, Vanderbilt University School of Medicine, Nashville, TN, USA; 4 Laboratory of Clinical Infectious Diseases, National Institutes of Health, Bethesda, MD, USA; 5 Center for Health Services Research, Department of Medicine, Vanderbilt University School of Medicine, Nashville, TN, USA

## Abstract

**Background:**

Approximately 5-10% of persons infected with *M. tuberculosis *develop tuberculosis, but the factors associated with disease progression are incompletely understood. Both linkage and association studies have identified human genetic variants associated with susceptibility to pulmonary tuberculosis, but few genetic studies have evaluated extrapulmonary disease. Because extrapulmonary and pulmonary tuberculosis likely have different underlying pathophysiology, identification of genetic mutations associated with extrapulmonary disease is important.

**Findings:**

We performed a pilot genome-wide association study among 24 persons with previous extrapulmonary tuberculosis and well-characterized immune defects; 24 pulmonary tuberculosis patients and 57 patients with *M. tuberculosis *infection served as controls. The Affymetrix GeneChip Human Mapping Xba Array was used for genotyping; after careful quality control, genotypes at 44,175 single nucleotide polymorphisms (SNPs) were available for analysis. Eigenstrat quantified population stratification within our sample; logistic regression, using results of the Eigenstrat analysis as a covariate, identified significant associations between groups. Permutation testing controlled the family-wise error rate for each comparison between groups. Four SNPs were significantly associated with extrapulmonary tuberculosis compared to controls with *M. tuberculosis *infection; one (rs4893980) in the gene PDE11A, one (rs10488286) in KCND2, and one (rs2026414) in PCDH15; one was in chromosome 7 but not associated with a known gene. Two additional variants were significantly associated with extrapulmonary tuberculosis compared with pulmonary tuberculosis; one (rs340708) in the gene FAM135B and one in chromosome 13 but not associated with a known gene. The function of all four genes affects cell signaling and activity, including in the brain.

**Conclusions:**

In this pilot study, we identified 6 novel variants not previously known to be associated with extrapulmonary tuberculosis, including two SNPs more common in persons with extrapulmonary than pulmonary tuberculosis. This provides some support for the hypothesis that the pathogenesis and genetic predisposition to extrapulmonary tuberculosis differs from pulmonary tuberculosis. Further study of these novel SNPs, and more well-powered genome-wide studies of extrapulmonary tuberculosis, is warranted.

## Introduction

Although approximately one-third of the world's population is infected with *M. tuberculosis*, [1] only 5-10% of infected individuals develop tuberculosis [2]. The primary site of disease is usually the lungs, but extrapulmonary sites may also be affected. Population-based surveys have found that 20% of tuberculosis cases are extrapulmonary [3]. With a global tuberculosis prevalence of 11.1 million cases,[4] this suggests a global prevalence of extrapulmonary tuberculosis of 2.2 million cases. Several factors are known to be associated with disease progression, including HIV infection, diabetes mellitus and malnutrition [5]. There is also evidence that some persons may have a genetic predisposition to tuberculosis [6-12]. Both linkage and association studies have identified candidate genetic variants and regions of the genome associated with tuberculosis risk [13-16], but they have focused primarily on pulmonary disease. Because the pathophysiology of pulmonary and extrapulmonary disease appears to differ [17,18], and because extrapulmonary disease in particular may be associated with an underlying immune defect [19-21], it is important to assess for genetic variants specifically associated with extrapulmonary tuberculosis.

Mutations in P2X7, [22] vitamin D receptor, [23] interleukin (IL) 1 - β, [24] LTA,[25] IL-10 [25], and IL-10 together with IFN-γ,[26-29] have been associated with extrapulmonary disease in previous candidate gene studies, and support the hypothesis of a genetic predisposition to extrapulmonary tuberculosis. We have previously confirmed the association between polymorphisms in the genes for vitamin D receptor and IL 1 - β and extrapulmonary disease, and reported a novel association between a polymorphism in the Toll-like receptor 2 gene with extrapulmonary tuberculosis, among black patients from the United States [30]. In this previous study, a candidate gene approach was utilized in which single nucleotide polymorphisms (SNPs) previously associated with tuberculosis as well as SNPs in candidate genes involved in tuberculosis pathogenesis were assessed. As a consequence, that study had limited potential to uncover novel genomic regions that play a role in the etiology of extrapulmonary tuberculosis. Rapid and affordable SNP genotyping across the entire genome is now possible, allowing for genome mapping to uncover novel associations not possible with a candidate gene approach. We are unaware of previous genome-wide association studies of extrapulmonary tuberculosis. We have performed a pilot genome-wide association study among HIV-seronegative persons in which the immunologic defects associated with extrapulmonary tuberculosis have been well-characterized, including low unstimulated cytokine production and low CD4+ lymphocyte levels [20,21,30].

## Methods

### Study participants

The study population was a pooled sample from two previous immunologic studies and the inclusion criteria for both studies have been described in detail previously [20,21,30]. Patients were selected through the Nashville Metropolitan Health Department Tuberculosis Clinic and the Baltimore City Health Department Eastern Chest Clinic, and all participants provided written informed consent. Extrapulmonary disease was defined as disease at any site outside of the pulmonary parenchyma. Eligibility criteria for extrapulmonary tuberculosis cases included: a history of completely treated culture-confirmed extrapulmonary tuberculosis, age ≥ 18 years, and HIV-seronegative status. Exclusion criteria for extrapulmonary tuberculosis cases included serum creatinine > 2 mg/dL, use of corticosteroids or other immunosuppressive agents at the time of diagnosis or time of study entry, malignancy, or diabetes mellitus. The inclusion criteria for pulmonary tuberculosis controls included a history of completely treated culture-confirmed pulmonary tuberculosis, age ≥ 18 years, no evidence of extrapulmonary tuberculosis, and HIV-seronegative status. Controls with latent *M. tuberculosis *infection were at least 18 years old, HIV-seronegative, and had a positive tuberculin skin test (purified protein derivative positive (PPD +), defined as ≥ 10 mm induration after intradermal placement of 5 tuberculin units of PPD) and without evidence of active tuberculosis. Participants in this control group were U.S.-born (and therefore not vaccinated with BCG), and were mostly close contacts of tuberculosis cases. The controls were sampled from the same two clinic populations as the cases, and were not related to the cases. Exclusion criteria for both control groups were the same as for cases.

### Laboratory Techniques

DNA was extracted from blood samples using the Puregene DNA Isolation kit (Gentra Systems, Minneapolis, MN) as per the manufacturer's protocol. Genomic DNA was stored at -70°C until genotyping. Laboratory personnel were blinded to the case-control status of the specimens.

### Genome-Wide DNA genotyping

All DNA samples underwent SNP genotyping using the GeneChip^® ^Human Mapping 50 K Xba Array (Affymetrix, Inc., Santa Clara, CA). The manufacturer's protocol was followed for all procedures. Briefly, a complexity reduction process was performed where genomic DNA (250 ng) was digested with *Xba*I, ligated to *Xba*I adaptor (Affymetrix), and amplified by polymerase chain reaction (PCR) using primers specific to the ligated adaptor. Cycling conditions were an initial denaturation of 94°C for 3 minutes followed by 30 cycles of 94°C for 30 seconds, 60°C for 45 seconds, and 68°C for 60 seconds. A final extension of 68°C for 7 minutes concluded the reactions. PCR products were assayed by gel electrophoresis, purified, fragmented to < 250 bp using dilute DNaseI (Affymetrix), biotin end-labeled with terminal deoxynucleotidyl transferase, and hybridized to the 50 K Xba Array at 48°C for 16 hours at 60 rpm. The hybridized arrays were washed and stained on Fluidics Station 450 and scanned with the GeneChip Scanner according to the manufacturer's settings (Affymetrix). The arrays were analyzed with software GDAS version 3.0.2 (Affymetrix), which provides rank scores for the probability of particular genotypes at SNP loci. The scores were AA or BB for homozygous alleles and AB for heterozygous alleles, and confidence scores showed the accuracy of the genotype call. Standard procedures and default analysis parameters for individual DNA samples were employed. An internal control run in parallel did not detect any DNA contamination. All procedures were performed using the same lots of reagents. Laboratory personnel were blinded to the case-control status of the specimen.

### Quality control

To prevent inclusion of genotyping errors in our association analysis, a 3-step filtering process was followed, which included: 1) removal of SNPs with < 90% genotyping efficiency (such that less than 10% of data were missing across all individuals in the dataset); 2) removal of individuals with genotyping efficiency < 90% (such that less than 10% of data were missing across all SNPs for each individual; 3) removal of SNPs out of Hardy-Weinberg equilibrium in PPD + controls (according to the results of a Fisher's Exact test) with p < 0.05 after Bonferroni correction for the total number of SNPs evaluated. PLINK version 1.04 [31] was used for quality control analysis and data processing. Of the 58,494 SNPs genotyped on the chip, 13,946 SNPs were removed for low genotyping rate (less than 90% of the participants could be genotyped for those SNPs). Each SNP was tested for deviation from Hardy-Weinberg Equilibrium using Fisher's exact tests in PPD + controls only. SNPs with p < 0.05 (after a Bonferroni correction for the number of tests performed) were removed (a total of 373), leaving 44,175 SNPs for statistical analysis. One PPD + control had < 90% genotyping efficiency and was removed from the analysis, leaving 104 individuals from the original 105.

### Statistical Analysis

The three patient groups (extrapulmonary tuberculosis, pulmonary tuberculosis, PPD +) underwent three comparisons for analysis: 1) extrapulmonary tuberculosis vs. PPD + controls to identify SNPs that are predictors of extrapulmonary disease, 2) extrapulmonary vs. pulmonary tuberculosis to identify differences between these two forms of disease, and 3) both extrapulmonary + pulmonary tuberculosis vs. PPD + controls to identify SNPs associated with active tuberculosis in general.

Clinical and demographic characteristics were compared among the three patient groups using the Kruskal-Wallis test for continuous variables and the chi-squared or Fisher exact tests for categorical variables, as appropriate based on distributional assumptions.

**Figure 1:**
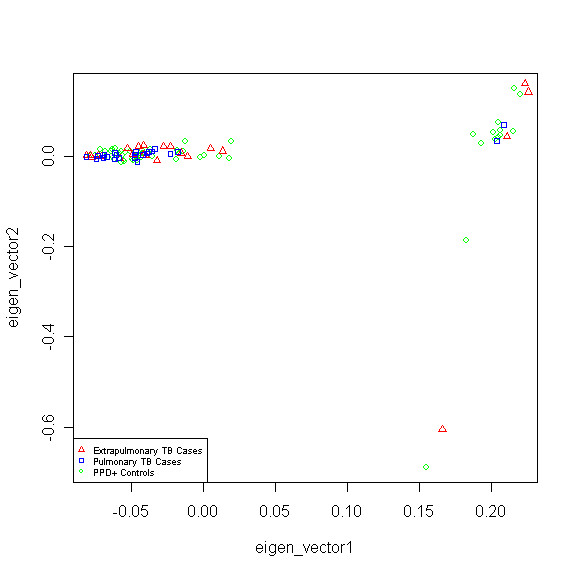
**Population Sub-structure Within our Study Population Using the Top Two Eigen Vectors Calculated by EIGENSTRAT**.

Population stratification, which refers to allele frequency differences between cases and controls due to ancestry, is an important concern in genetic association tests, as it can cause spurious associations [32-34]. To address this problem in our diverse patient population the EIGENSTRAT method, which uses principal component analysis (PCA), was used to quantify and correct for population sub-structure and adjust for population stratification in association analyses [35,36]. The EIGENSTRAT utility of the EIGENSOFT package version 2.0 was used for our analysis [36]. The method infers axes of variation to reduce the dimensionality of the data while describing as much variability as possible. We used EIGENSTRAT to evaluate the top ten Eigen vectors of the covariance matrix between the samples [36]. Figure 1 shows the first two axes of variation from the PCA analysis. The results revealed two major clusters within our population and a third small cluster defined by a third axis of variation. These three groups (in order) were consistent with the self-reported racial groups of our sample (black, white, and Asian). These three clusters were used as categorical covariates in the genetic association analysis.

PLINK version 1.04 [31] was used for association analyses. Logistic regression using the cluster definitions from the EIGENSTRAT analysis as a covariate in each regression model, was used to evaluate potential associations for each SNP, for each of the three comparisons described above (using a genotypic encoding for the SNP variables). Dummy encoding was used for each SNP variable, such that no genetic model assumptions were made in the analysis. WGA Viewer [37] was used for the graphical representation of our data as well as for the selection of the SNPs with the strongest evidence for association. Annotation of SNPs was then done using the Ensemble database release 59 [38].

A major concern in genetic association studies in general, and genome-wide association studies in particular, are false positive findings due to multiple comparisons [39,40]. When evaluating thousands of potential associations, at a nominal alpha rate of 0.05, 5% of the associations may be statistically significant by chance alone. To decrease the false positive rate of the current study, permutation testing of all 44,175 SNPs as a set [41] was used to derive empirical cut-offs that corresponded to a family-wise (experiment wise) error rate of 0.05 for each comparison. This less stringent empirical p-value was used instead of the more widely used genome-wide significance threshold of 5 × 10^-8 ^due to the small sample size of our study. Permutation testing was implemented in PLINK, and SNPs in each comparison with raw p-values below the empirical p-value cut-off derived from permutation testing corresponding to a family-wise error rate of 0.05 for each of the three comparisons were considered statistically significant. To check whether the association signals that pass this significance threshold are due to linkage disequilibrium (LD), r^2 ^scores for SNPs on the same chromosome and/or gene were also calculated. The overall level of heterozygosity (Fst) in our sample was also calculated using GENEPOP version 1.2 [42].

## Results

There were 44,175 SNPs and 104 individuals for analysis after applying our quality control filter: 24 extrapulmonary tuberculosis cases, 24 pulmonary tuberculosis controls, and 56 PPD + controls. The sites of extrapulmonary disease have been reported previously [30]; only 1 had central nervous system (miliary/meningeal) disease. The clinical and demographic characteristics of the 104 patients in the study population are in Table 1. There were no statistically significant differences by age, sex, or race between cases and controls.

**Table 1:** Clinical and Demographic Characteristics of the Study Population

Characteristic	Extrapulmonary TB (n = 24)	Pulmonary TB (n = 24)	PPD + (n = 56)	**P-value**^**a**^
Median age at study entry in years (IQR)	48.4 (43.6-79.4)	43.6 (40.3-53.9)	46 (39.3-53.5)	0.19
# Male Sex (%)	16 (67)	14 (58)	35 (63)	0.87
# Black Race (%)	18 (75)	19 (79)	48 (86)	0.16
# White Race (%)	4 (17)	4 (17)	8 (14)	0.27
# Asian Race (%)	2 (9)	1 (4)	0 (0)	0.22

IQR: inter-quartile range

**Figure 2:**
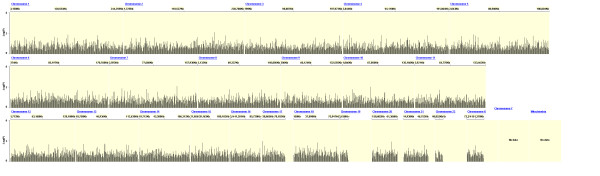
**Distribution of p-values from the Logistic Regression Test of Extrapulmonary Tuberculosis Cases vs. PPD + Controls**.

**Figure 3:**
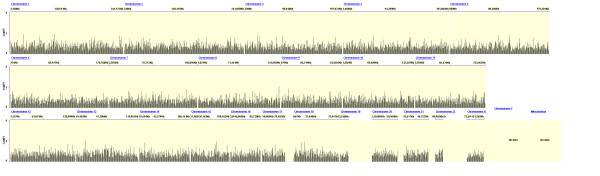
**Distribution of p-values from the Logistic Regression Test of Extrapulmonary Tuberculosis Cases vs. Pulmonary Tuberculosis Controls**.

**Figure 4:**
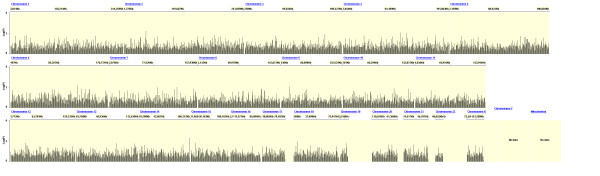
**Distribution of p-values from the Logistic Regression Test of all Tuberculosis Patients (Pulmonary and Extrapulmonary) vs. PPD + Controls**.

**Figure 5:**
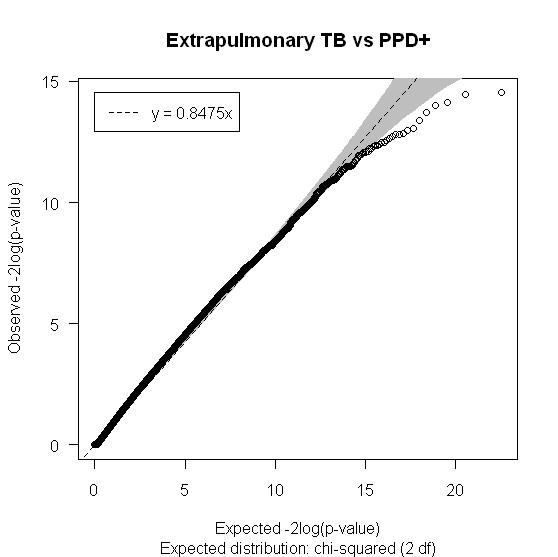
**Q-Q plot of the expected versus observed -2log(p-values) for Extrapulmonary TB vs PPD +**.

**Figure 6:**
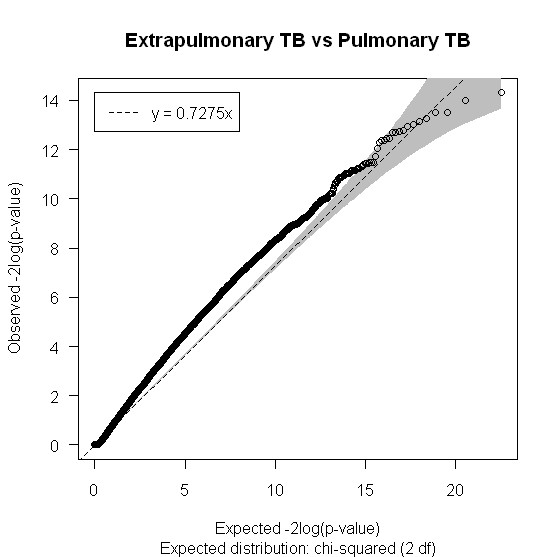
**Q-Q plot of the expected versus observed -2log(p-values) for Extrapulmonary TB vs Pulmonary TB**.

**Figure 7:**
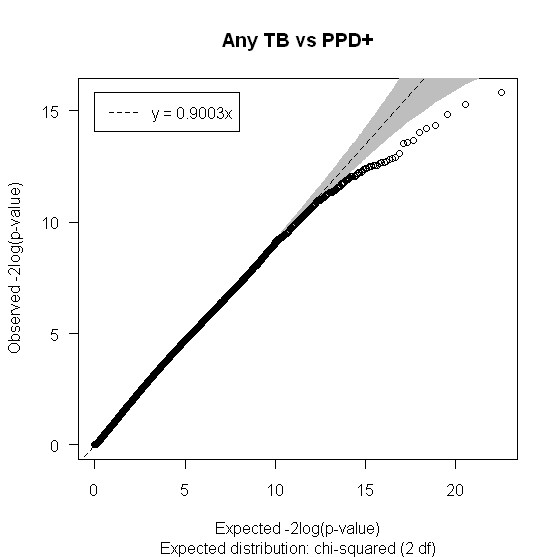
**Q-Q plot of the expected versus observed -2log(p-values) for Any TB vs PPD +**.

Figures 2, 3 and 4 show the distribution of SNPs and the -log(p) values for each SNP from the logistic regression analysis for the three comparisons (extrapulmonary tuberculosis vs PPD +, extrapulmonary tuberculosis vs pulmonary tuberculosis and any tuberculosis vs PPD +, respectively), as well as the coverage achieved using the Affymetrix 50 k array. The empirical P-value cutoff from permutation testing was 0.0009. Figures 5, 6 and 7 show Q-Q plots demonstrating the expected versus observed -2log(p-values) for each comparison: extrapulmonary tuberculosis vs PPD +, extrapulmonary tuberculosis vs pulmonary tuberculosis and any tuberculosis vs PPD +, respectively. These Q-Q plots show the observed versus expected p-values under the null hypothesis. A large number of p-values above the diagonal line would indicate strong signal within the data (especially at the top right portion of the plot), whereas a large number of p-values below the diagonal line indicate low statistical power. Deviation of the observed p-values from the diagonal across the entirety of the plot (not just at the top right) indicate possible technical concerns with the study. Our results demonstrate that there are not major technical concerns, but statistical power is low. Figure 6 demonstrates slight deviation from the diagonal, but generally all comparisons show overall good quality. These findings should be kept in mind when interpreting the results of this pilot study. The level of genetic variation between the two types of active disease (pulmonary and extrapulmonary tuberculosis) and PPD + controls was calculated using EIGENSTRAT [35] and was found to be 0.001. This demonstrates a low level of variability between those with prior tuberculosis disease and the PPD + group, indicating little differentiation among the groups, after accounting for racial differences.

### Extrapulmonary tuberculosis vs. PPD + controls

There were four statistically significant SNPs between these two patient groups, three of which are in known genes: PDE11A, KCND2 and PCDH15 (Table 2). Two of these SNPs (rs10487416 and rs10488286) were on the same chromosome (chromosome 7) and the LD scores for these two SNPs on chromosome 7 are in Table 3.

**Table 2:** Statistically Significant SNPs Identified in Extrapulmonary Tuberculosis Cases vs. PPD + Controls

SNP	Rank	P-value	MAF in ETB	MAF in PPD +	Odds Ratio	Chromosome	Gene	Closest Gene	Distance to Gene
rs4893980	1	0.0007	0.1087	0.4902	0.1268	2	PDE11A	---	---
rs10487416	2	0.0007	0.25	0.0566	5.556	7	unknown	unknown	unknown
rs10488286	3	0.0009	0.2381	0.02727	11.15	7	KCND2	---	---
rs2026414	4	0.0009	0.5208	0.2589	3.111	10	PCDH15	---	---

Empirical P - value cutoff from permutation testing = 0.0009

The p - value reported is the raw value.

MAF = Minor Allele Frequency

ETB = Extrapulmonary tuberculosis

PPD + = Purified protein derivative positive

**Table 3:** Linkage Disequilibrium (LD) scores of Statistically Significant SNPs Identified in the Same Gene or chromosome

chromosome	Comparison	SNP1	SNP2	D'	LOD	r^2^
7	ETB vs PPD +	rs10487416	rs10488286	0.406	3.08	0.148
4	Any TB vs PPD +	rs2107005	rs509423	0.345	0.95	0.043

PPD + = Purified protein derivative positive

ETB = Extrapulmonary tuberculosis

Any TB = Pulmonary or extrapulmonary tuberculosis

### Extrapulmonary tuberculosis vs. pulmonary tuberculosis

There were two SNPs that were significantly different between these two groups (Table 4), and the most significant one (lowest p-value) was in the gene FAM135B. The two SNPs were on different chromosomes so no LD calculations were made for this subgroup.

**Table 4:** Statistically Significant SNPs Identified in Extrapulmonary Tuberculosis Cases vs. Pulmonary Tuberculosis Controls

SNP	Rank	P-value	MAF in ETB	MAF in PTB	Odds Ratio	Chromosome	Gene
rs340708	1	0.0008	0.4375	0.125	5.444	8	FAM135B
rs1886870	2	0.0009	0.6667	0.25	6	13	RP11-141M1

Empirical P-value cutoff from permutation testing = 0.0009

The p-value reported is the raw value.

MAF = Minor Allele Frequency

ETB = Extrapulmonary tuberculosis

PTB = Pulmonary tuberculosis

### Any tuberculosis vs. PPD + controls

Six SNPs were significantly different in this comparison (Table 5), of which five (rs1989565, rs10490266, rs2107005, rs509423, rs968079) were in known genes: MARK3, AC007317.1, RP11-556L14.2, RP11-440N4 respectively, and SNP rs1518350 was not in any know gene but was closest to the gene SLC35F1. Two SNPs (rs2107005 and rs509423) were on the same chromosome (chromosome 4), and their LD scores are in Table 3.

**Table 5:** Statistically Significant SNPs Identified in Patients with Any Tuberculosis (Pulmonary or Extrapulmonary) vs. PPD + Controls

SNP	Rank	P-value	MAF in any TB	MAF in PPD +	Odds Ratio	Chromosome	Gene	Closest Gene	Distance to Gene
rs1989565	1	0.0004	0.1702	0.4074	0.2984	14	MARK3	---	---
rs10490266	2	0.0005	0.125	0.3661	0.2474	2	AC007317.1		
rs1518350	3	0.0006	0.4688	0.2143	3.235	6	---	SLC35F1	29,253bp
rs2107005	4	0.0008	0.2917	0.5804	0.2977	4	RP11-556L14.2	---	---
rs509423	5	0.0008	0.1064	0.3039	0.2727	4	RP11-440N4	---	---
rs968079	6	0.0009	0.587	0.3163	3.071	9	PTPRD	---	---

Empirical P - value cutoff from permutation testing = 0.0009

The p - value reported is the raw value

MAF = Minor Allele Frequency

TB = Pulmonary or extrapulmonary tuberculosis

PPD + = Purified protein derivative positive

## Discussion

The results of this pilot study reveal 6 SNPs that were significantly associated with extrapulmonary tuberculosis: four SNPs distinguished between extrapulmonary tuberculosis and *M. tuberculosis *infection and two distinguished between extrapulmonary and pulmonary tuberculosis. There were an additional 6 SNPs that distinguished between active tuberculosis of any site (pulmonary or extrapulmonary) and *M. tuberculosis *infection. None of the SNPs identified have previously been reported to be associated with tuberculosis. This is to be expected since almost all of the SNPs included in the Affymetrix 50K Xba chip have not previously been reported to be associated with tuberculosis risk, nor in genes known to be important in tuberculosis pathogenesis. Most of the SNPs were found in regions with newly discovered genes of which very little is known, as such, this study should be viewed as exploratory and a way to uncover potentially novel biology. The SNPs identified should be evaluated in subsequent studies of tuberculosis genetics, and their immunologic correlate should be assessed.

The analysis of persons with extrapulmonary tuberculosis vs. PPD + controls (Table 2) identified 4 SNPs that were significantly associated with extrapulmonary disease. Three of the SNPs were in known genes (PDE11A, KCND2, PCDH15). While much is known about the function of these genes themselves, how they may potentially play a role in tuberculosis pathogenesis is unclear. None of these genes have previously been reported to be associated with tuberculosis. PDE11A encodes a member of the PDE superfamily, which plays a role in cAMP and cGMP function as second messengers in a wide variety of signal transduction pathways. Mutations in this gene are a cause of Cushing disease and adrenocortical hyperplasia [43]. The gene also appears to play a significant role in regulating brain function. KCND2 encodes a member of the family of voltage-gated potassium channels (shal-related sub-family) which is prominent in the repolarization phase of the action potential [44]. The diverse functions of these channels include regulating neurotransmitter release, heart rate, insulin secretion, neuronal excitability, epithelial electrolyte transport, smooth muscle contraction, and cell volume. PCDH15 is a member of the cadherin superfamily. Family members encode integral membrane proteins that mediate calcium-dependent cell-cell adhesion. It plays an essential role in maintenance of normal retinal and cochlear function. Mutations in this gene result in hearing loss and Usher Syndrome Type IF [45].

It has been previously suggested that the pathogenesis of pulmonary tuberculosis may differ from that of extrapulmonary tuberculosis. While this study was under-powered to fully evaluate differences in genetic predisposition, the results from the extrapulmonary tuberculosis vs. pulmonary tuberculosis (Table 4) analysis provides some evidence in support of this hypothesis.

Two SNPs were significantly associated with extrapulmonary vs. pulmonary tuberculosis, one of which was in a known gene FAM135B (family with sequence similarity 135, member B). The gene FAM135B located on chromosome 8 and is a protein coding gene that is expressed in several tissues/organs such as the brain and heart, but has not previously been linked to either form of tuberculosis. The other significant polymorphism (rs1886870) for this comparison is in a newly annotated gene RP11-141M1 of which not much is currently known.

The level of linkage disequilibrium between significant SNPs found on the same gene and those on the same chromosome was calculated: the low correlation coefficient scores (r^2^) suggest that the SNPs are independent of each other (Table 3).

The biological relevance of the SNPs identified is difficult to determine because there is very little known about these SNPs. Most were in unknown regions of the genome and those that were found within gene regions have not been associated with either extrapulmonary or pulmonary tuberculosis. Additional study is warranted to assess the possible biological relevance.

Our findings from the third comparison (both forms of tuberculosis vs PPD + controls) identified 6 SNPs associated with tuberculosis risk (Table 5). One of these SNPs (rs1989565) was in the MARK3 (microtubule affinity-regulating kinase 3) gene, which is a protein coding gene that might play a role in the regulation of cell polarity [46]. This gene has not previously been linked with tuberculosis risk. Another SNP (rs968079) was found to be in the gene PTPRD (protein-tyrosine phosphatase receptor-type delta) which has been associated with asthma[47], Restless leg syndrome[48] and some cancers as a tumor suppressor[49]. Three SNPs (rs2107005, rs509423, rs10490266) were found in three newly annotated genes and not much is known about them. The final significant SNP in this comparison was not found in any gene but was closest to the gene SLC35F1(Solute Carrier Family 35, Member F1), which is mainly expressed in the brain[50], but not much else is known about its function.

Though our sample size was small we controlled for population stratification to reduce the occurrence of spurious results; such an approach has also been shown to increase power [51]. Additionally, we used permutation testing to control for false positive findings. While the current findings would not hold up to a stringent Bonferroni correction for genome-wide significance, the permutation testing does control the family-wise error rate for these tests.

It is clear that the major limitation of this study was the sample size, which leaves us under-powered to detect small effects as shown by our Q-Q plots (Figures 5, 6 and 7), and therefore only very large effect sizes could be detected. Although we did not have the power to detect potentially important genetic predictors of tuberculosis risk, the SNPs identified were by necessity, strong predictors. They should also be interpreted within the context of the immunologic deficits noted in these patients with previous extrapulmonary tuberculosis [20,21]. Our results should be considered hypothesis generating, as opposed to hypothesis testing, and should be evaluated in subsequent studies of extrapulmonary tuberculosis with substantially increased sample sizes.

A second limitation was the limited SNP coverage of the Affymetrix 50 k array. In particular, large regions in chromosomes 1, 9 and 16 were not covered. Newer arrays, with a greater number of SNPs and more extensive coverage, would potentially identify even more novel SNP associations. However, the above issues of sample size, statistical power, and multiple comparisons would take on even greater importance.

## Conclusions

Novel genetic factors were associated with susceptibility to extrapulmonary tuberculosis, and these factors may act through previously unknown pathophysiologic mechanisms. The genes identified play a role in cellular signaling, activity and polarity, with some appearing to play an important role in the brain. Only one of the patients with extrapulmonary disease had central nervous system involvement. Future large-scale studies are warranted in which persons with extrapulmonary tuberculosis and appropriate controls have both genetic and immunologic features characterized. Additional studies of the biologic function of the SNPs identified here would also provide additional insight into the pathophyisology of extrapulmonary tuberculosis.

## Abbreviations

*M. tuberculosis*: *Mycobacterium tuberculosis; *TB: tuberculosis; ETB: extrapulmonary tuberculosis; PTB: pulmonary tuberculosis; PPD: purified protein derivative; SNP: single nucleotide polymorphism; MAF: minor allele frequency;

## Competing interests

None of the authors have a commercial or other association that might pose a conflict of interest related to this work.

## Authors' contributions

NOO led the analysis and writing of the manuscript. AAMR contributed to the analysis and writing of the manuscript, PRZA contributed to the design of the study, preparation of the samples to be tested for polymorphisms and also to the methods section of the manuscript. SL oversaw the performance of the genotyping. SMH contributed to the design of the study. TRS conceived and designed the study and made significant contributions to the writing of the manuscript. The final manuscript was read and approved by all authors.
